# Site-to-site mutational dissection of fission yeast cohesin reveals its dynamics

**DOI:** 10.1093/g3journal/jkaf111

**Published:** 2025-05-19

**Authors:** Qi Wei, Li Wang, Yichen Zhang, Saidaiguli Abulimiti, Jie Wang, Xingya Xu

**Affiliations:** Institute of Future Agriculture, Northwest A&F University, Yangling, Shaanxi 712100, People's Republic of China; College of Enology, Northwest A&F University, Yangling, Shaanxi 712100, People's Republic of China; Institute of Future Agriculture, Northwest A&F University, Yangling, Shaanxi 712100, People's Republic of China; Institute of Future Agriculture, Northwest A&F University, Yangling, Shaanxi 712100, People's Republic of China; Institute of Future Agriculture, Northwest A&F University, Yangling, Shaanxi 712100, People's Republic of China; Institute of Future Agriculture, Northwest A&F University, Yangling, Shaanxi 712100, People's Republic of China

**Keywords:** fission yeast, chromosome segregation, cohesin, suppressor screen, cell cycle

## Abstract

Cohesin is a heteropentameric protein complex that holds sister chromatids together from S phase to anaphase. Its 2 structural maintenance of chromosome subunits form a heterodimer, consisting of an ATPase head domain and a hinge domain connected by long coiled coils. Kleisin subunit associates with the head. Here, using *Schizosaccharomyces pombe*, we genetically dissected cohesin dynamics based on the relationship between the mutations causing temperature-sensitive and their suppressor mutations. First, we identified suppressor mutations that could rescue the lethality caused by cohesin ATPase mutations. Mutations in the DNA-binding domain of cohesin loader Mis4, or in cell-cycle genes encoding MBF transcription factor complex or Wee1 kinase, rescued both Psm1 and Psm3 ATPase mutants. Then, we performed targeted mutagenesis in both ATPase domains for single-amino-acid substitutions that can rescue the lethality of a kleisin ts mutant at restrictive temperature. Comparison of mutations obtained in Psm1 and Psm3 ATPase domains revealed that analogous mutations in the 2 ATPase domains were frequently observed. Last, suppressors of a coiled-coil mutation were mapped in coiled coils, indicating that proper folding of coiled coils is critical for cohesin functions. Suppressors of a hinge interface mutation are frequently located at the other hinge interface, indicating that the 2 cohesin hinge interfaces work collaboratively in hinge–hinge interactions. Overall, genetic dissection of the relationship between cohesin lethal mutations and their suppressor mutations reflects cohesin dynamics in vivo.

## Introduction

Cohesin is a multi-subunit protein complex that plays critical roles in sister chromatid cohesion, DNA damage response, genome spatial organization, and gene expression regulation (Uhlmann 2016; Yatskevich et al. 2019). Cohesin complex consists of 5 subunits: Psm1/SMC1, Psm3/SMC3, Rad21/Kleisin, Mis4/SCC2/NIPBL, and Psc3/STAG1–3. All of these subunits are essential in fission yeast *Schizosaccharomyces pombe* (*S. pombe*) as deletion of either of these genes is not viable (Tomonaga et al. 2000). Psm1 and Psm3 serve as heterodimeric structural maintenance of chromosome (SMC) subunits. The 3 non-SMC subunits associate with cohesin head domain or the immediate coiled coils close to the head domain (Haering et al. 2004; Gligoris et al. 2014; Huis in ‘t Veld et al. 2014; Higashi et al. 2020) (Supplementary Fig. 1a). Mis4, which functions as the cohesin loader that deposits cohesin onto chromatin in G1/S phase (Furuya et al. 1998), forms a harp-shaped structure and exhibits DNA-binding activity in vitro (Murayama and Uhlmann 2014; Kikuchi et al. 2016; Chao et al. 2017).

Both Psm1 and Psm3 monomers contain an N-terminal domain, a central hinge domain and a C-terminal domain (Supplementary Fig. 1b). These SMC proteins fold back at the hinge, forming antiparallel coiled coils that are about 50 nm in length. The N-terminal domain and the C-terminal domain come together to form the globular head. Psm1 and Psm3 form a heterodimer. Atomic force microscopy (Sakai et al. 2003; Bauer et al. 2021), electron microscopy (Anderson et al. 2002; Huis in ‘t Veld et al. 2014), and structural analysis (Haering et al. 2002; Haering et al. 2004) have demonstrated that, within the SMC dimer, hinge domains interact to form a donut-shaped structure and each SMC protein's N-terminal domain binds the other SMC protein's C-terminal domain to form an ATPase domain. Each ATPase domain binds 1 ATP molecule, which can be hydrolyzed by the ATPase domain. Each ATPase domain contains the Walker A (Wal-A) and Walker B (Wal-B) consensus sequences found in most ATPases (Lammens et al. 2004). In addition to the Wal-A and Wal-B motifs, 6 unique sequence motifs were found in each ATPase domain; they are A-loop, Q-loop, C-motif, C-helix, D-loop, and H-loop. A-loop, Wal-A, and Q-loop locate at SMC protein's N-terminal domain, while C-motif, C-helix, Wal-B, D-loop, and H-loop locate at SMC protein's C-terminal domain (Supplementary Fig. 1, c and d). ATP hydrolysis is required for cohesin to associate with chromosomes (Arumugam et al. 2003). In vitro biochemical studies demonstrated that addition of cohesin loader Mis4/SCC2/NIPBL and DNA greatly stimulated the ATPase activities of cohesin (Kim et al. 2019).

The donut-shaped structure of hinge contains 2 (north and south) interaction interfaces (Haering et al. 2002). Each hinge dimerization interface contains a conserved arrangement of glycine residues (GX6GX3GG) that are essential for dimer stability (Hirano et al. 2001; Hirano and Hirano 2002, 2006). Both interaction interfaces are essential for cohesin's stable association with chromosomes (Mishra et al. 2010). Substitution of the conserved glycine residues at the interaction interfaces frequently caused sensitivity to cold (20°C), sensitivity to DNA damaging agents, and chromosome mis-segregation at restrictive temperature (Xu et al. 2018; Xu and Yanagida 2019a).

Multiple cohesin mutants, carrying single-amino-acid substitutions that impair specific functions of cohesin, have been well characterized, and suppressor mutations that restored the defective functions of cohesin were also identified (Xu et al. 2018; Xu and Yanagida 2019b; Xu et al. 2022a). The relationship between the original ts/cs mutations and their suppressor mutations effectively illustrated cohesin's structural organization and dynamics (Xu and Yanagida 2023). Among these studies, unbiased screening of spontaneous suppressor mutations, which rescued the temperature lethality of cohesin ATPase mutants, identified several suppressor hotspots in cohesin that are located outside of ATPase domains in Psm1 and Psm3 (Xu et al. 2022a). Here, by using the same cohesin ATPase mutants, we identified many extragenic suppressor mutations in cohesin loader Mis4 and cell cycle regulators (Cdc10, Res2, and Wee1). Moreover, we identified suppressor mutations of a kleisin mutant, *rad21-I67F*, in cohesin ATPase head domains; suppressor mutations of a coiled-coil mutant, *psm3-304*, in cohesin coiled coils; and suppressor mutations of a hinge mutant, *psm3-G653E*, in cohesin hinge domains. The genetic dissection provides valuable clue to understand cohesin organization and dynamics in vivo.

## Materials and methods

### Strains, plasmids, and media

Parental *S. pombe* ts/cs strains of *psm1*, *psm3*, and *rad21* have been described previously (Xu et al. 2018; Xu and Yanagida 2019b; Xu et al. 2022a). YPD (1% yeast extract, 2% polypeptone, 2% D-glucose) were used to culture the *S. pombe* strains.

### Suppressor screen and mutation identification

Suppressor screen, next-generation sequencing, and mutation identification followed the suppressor screening protocols described in the previous studies (Xu et al. 2018; Xu and Yanagida 2019b; Xu et al. 2022a).

### Site-directed saturation mutagenesis

The *psm1-L1166N*, *psm3-S1098A*, and *rad21-I67F* ts mutants, and the *psm3-G653E* cs mutant isolated in the previous studies (Xu et al. 2018; Xu and Yanagida 2019b; Xu et al. 2022a), were used as the host strains for site-directed saturation mutagenesis. The targeting vectors of Mis4, Psm1, and Psm3, which were based on the pBluescript plasmid containing a nourseothricin sulfate (or clonNAT) resistant antibiotic marker, were used as PCR templates for saturation mutagenesis. A site-directed PCR-based mutagenesis, according to the method described in a previous study (Xu et al. 2022b), was then performed to introduce random “NNN” (encoding 1 amino acid) into the wild-type ORFs of Mis4/Psm1/Psm3 to substitute 1 amino acid in the selected sequence motifs. In total, 50 such PCR-based mutagenesis experiments for Mis4, 51 such PCR-based mutagenesis experiments for Psm1, and 51 such PCR-based mutagenesis experiments for Psm3 were performed. A mutation library for Mis4/Psm1/Psm3 was generated by mixing equal amount of each PCR product together. The mutation libraries were then transformed into the corresponding ts/cs mutants, respectively, spread onto YPD plates containing clonNAT (200 μg/mL), and incubated at restrictive temperatures for 6∼8 days. Revertants were then picked up. Targeted sequencing was performed to identify suppressor mutation in each revertant.

### Phenotypic observation

Cells were cultured to 0.6∼1 × 10^7^ cells per milliliter in YPD medium, fixed with 2% glutaraldehyde for 30 min on ice, stained with DAPI (4,6-diamidino-2-phenylindole, a fluorescent probe for DNA), and then observed under a spinning disk confocal system (Nikon CSU-W1).

### Prediction of coiled-coil probabilities

The protein sequences of wild-type Psm1, Psm3, and Cut14 were downloaded from PomBase database (https://www.pombase.org/) (Wood et al. 2012). Then coiled-coil probabilities of wild-type Psm1, Psm3, and Cut14 were calculated using MARCOIL program (Delorenzi and Speed 2002), integrated in the MPI Bioinformatics Toolkit (https://toolkit.tuebingen.mpg.de/tools/marcoil) (Biegert et al. 2006).

## Results and discussion

### Mutations in Mis4 DNA-binding domain rescue cohesin ATPase mutants

**Fig. 1.**
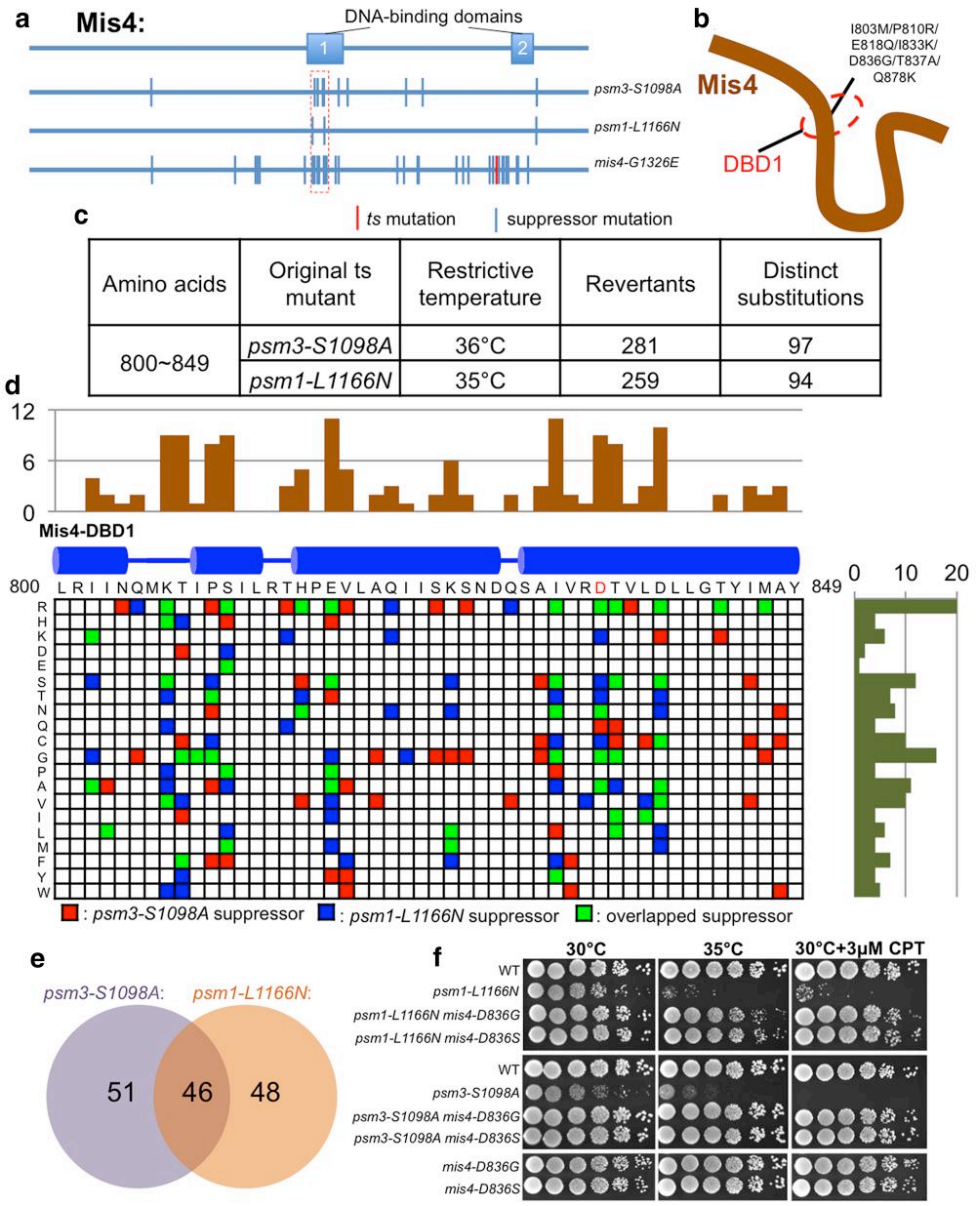
Suppressors of cohesin ATPase ts mutants mapped in Mis4. a) Localization of suppressor mutations of *psm1-L1166N*, *psm3-S1098A*, and *mis4-G1326E* identified in Mis4. Amino acids: 800∼849 were highlighted by a dashed rectangle. b) Suppressor mutations located in the DBD 1 (Mis4-DBD1; amino acids: 800∼849) of Mis4. c) Summary of the targeted mutagenesis for identification of single-amino-acid substitutions that can rescue the temperature sensitivity of the cohesin ATPase ts mutants (*psm1-L1166N* and *psm3-S1098A*). d) The single-amino-acid substitutions (the colored squares in the matrix) identified as suppressors of the *psm1-L1166N* and/or *psm3-S1098A* ts mutants. e) Mutational overlap between the *psm1-L1166N* suppressors and the *psm3-S1098A* suppressors in Mis4-DBD1. f) Spot test analysis.

Two temperature-sensitive (ts) mutants, carrying single-residue substitutions at key positions in cohesin's 2 ATPase sites, were previously isolated and characterized (*psm1-L1166N* and *psm3-S1098A*) (Xu et al. 2022a). Extragenic suppressor identification using next-generation sequencing found multiple mutations in cohesin loader Mis4, particularly within 1 of its 2 DNA-binding domains (DBDs) (Fig. 1, a and b; Supplementary Table 1a). To better understand the genetic suppression, we designed serial targeted mutagenesis, which introduce “NNN” random codon aiming to mutate the targeted amino acid to any of the other 19 amino acids (or stop codons). In total, 50 amino acids in Mis4 DBD1 (AA: 800∼849) were covered. Then, we isolated revertants that can grow at restrictive temperature (36°C for *psm3-S1098A* and 35°C for *psm1-L1166N*), after transforming the Mis4-DBD1 mutation pools into the cohesin ATPase mutants (*psm1-L1166N* and *psm3-S1098A*). A total of 281 and 259 revertants of *psm3-S1098A* and *psm1-L1166N* were analyzed, respectively, by targeted sequencing of the Mis4-DBD1 region. Finally, 97 and 94 single-amino-acid substitutions in Mis4-DBD1 were identified (Fig. 1c). The single-amino-acid substitutions were presented in a matrix (Fig. 1d). Many substitutions were identified as suppressors of both *psm3-S1098A* and *psm1-L1166N* (Fig. 1, d and e). To verify the genetic suppression, we independently introduced 2 suppressor mutations (*mis4-D836G* and *mis4-D836S*) into *psm1-L1166N* and *psm3-S1098A* ts mutants. Spot tests confirmed the genetic suppression well (Fig. 1f).

**Fig. 2.**
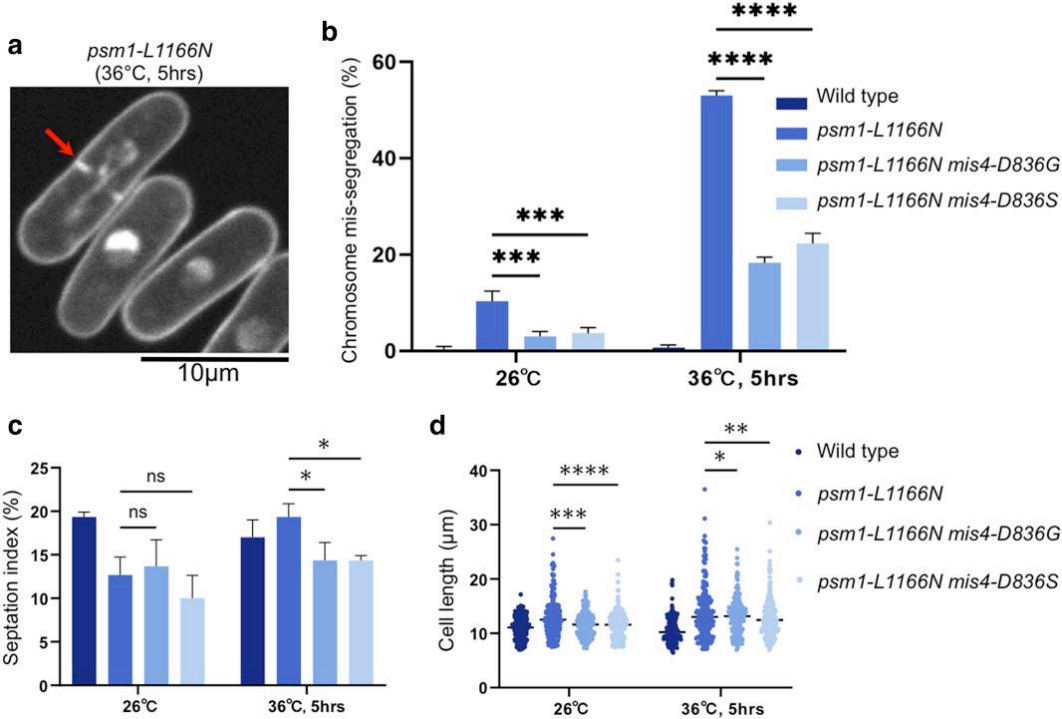
Phenotypic suppression of a cohesin ATPase mutant by mutations in Mis4. a) DAPI staining followed by microscopic observation for *psm1-L1166N* mutant cells cultured at restrictive temperature 36°C for 5 h. A cell with chromosome mis-segregation was indicated by an arrow. b) Frequency of chromosome mis-segregation events calculated by counting 200 mitotic cells. c) Septation index calculated by counting number of cells having septation in 200 cells. d) Cell length was measured using the ImageJ software.

Chromosome mis-segregation was frequently observed in *psm1-L1166N* (and *psm3-S1098A*) when cultured at restrictive temperature (Fig. 2a). Frequency of the chromosome mis-segregation events decreased dramatically when suppressor mutation in Mis4-DBD1 (*mis4-D836G* or *mis4-D836S*) was introduced into the *psm1-L1166N* mutant (Fig. 2b). Additionally, *psm1-L1166N* cells exhibited elongated morphology compared to wild-type cells, which indicated that cell cycle progression might be problematic in *psm1-L1166N* at restrictive temperature. This elongation phenotype was rescued by introduction of suppressor mutations in Mis4-DBD1 (either *mis4-D836G* or *mis4-D836S*) into the *psm1-L1166N* mutant (Fig. 2, c and d).

Since the cohesin loader Mis4 binds to DNA via its 2 DBDs (Kurokawa and Murayama 2020), the suppressor mutations of cohesin ATPase ts mutants, which were enriched in the 2 DBDs of Mis4, might affect the DNA-binding ability of Mis4. Among the suppressors of cohesin ATPase ts mutants, arginine (R) was most frequently observed in mutant alleles (Fig. 1d). Substitutions to arginine might enhance the DNA-binding ability of Mis4-DBD by establishing new electrostatic interactions with chromosomal DNAs.

### Mutations in MBF or Wee1 genes rescue cohesin ATPase mutants

**Fig. 3.**
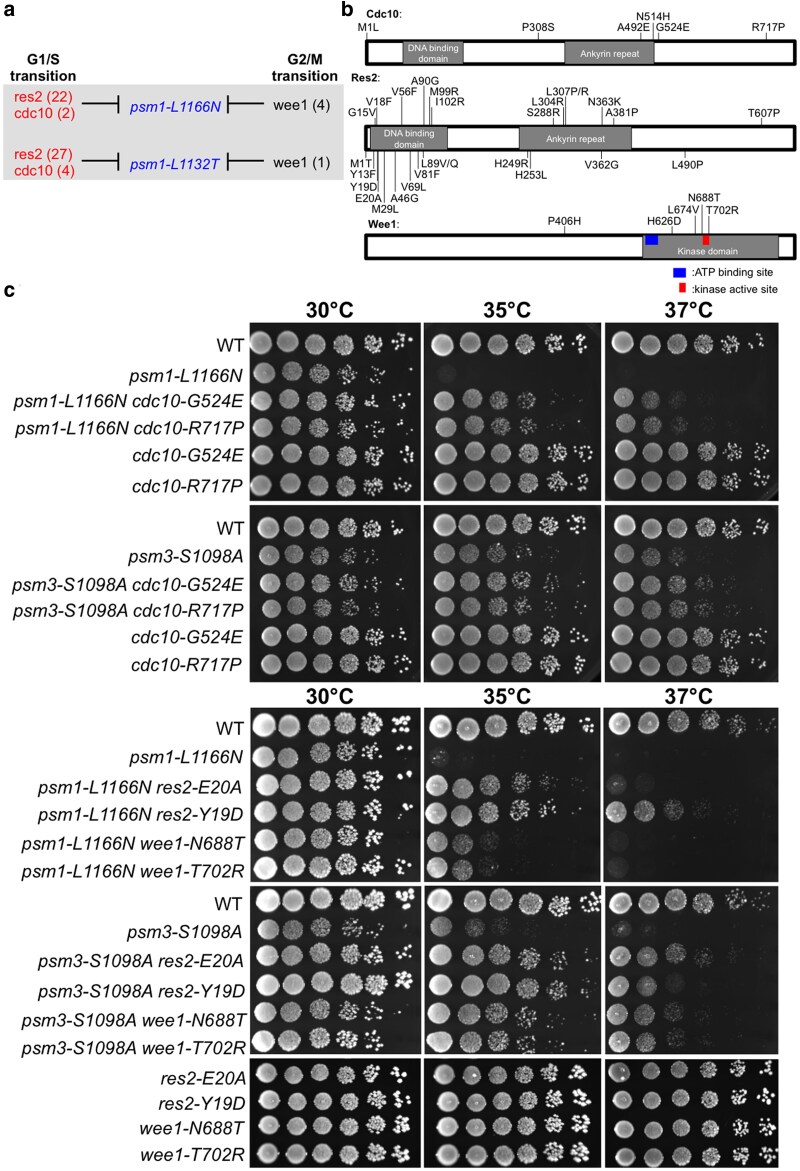
Suppressors of cohesin ATPase mutants in Res2-Cdc10 and Wee1. a) Suppressor mutations of the *psm1-L1166N* and *psm3-S1098A* mutants were identified in *res2*, *cdc10*, and *wee1*. b) Mutation localizations in Cdc10, Res2, and Wee1. Suppressor mutations of *psm1-L1166N* in Res2 were shown above the Res2 protein sequence, while suppressor mutations of *psm3-S1098A* in Res2 were shown below the Res2 protein sequence. c) Spot test analysis.

Extragenic suppressors of cohesin ATPase ts mutants (*psm1-L1166N* and *psm1-L1132T*) were mapped in cell cycle genes (*cdc10*, *res2*, and *wee1*) too (Fig. 3a; Supplementary Table 1, b–d). Res2 and Cdc10 form Res2-Cdc10 complex, and it functions in G1 phase to activate genes essential for the onset and progression of S phase, whereas Wee1 kinase regulates the G2/M transition. Mutations' localizations were analyzed according to protein sequences. Cdc10 and Res2 mutations were enriched in their DBD and/or ankyrin repeat domain, while Wee1 mutations were enriched in its C-terminal kinase domain (Fig. 3b). Re-introduction of the *cdc10*, *res2*, and *wee1* mutations into *psm1-L1166N* confirmed the genetic suppression. Furthermore, these mutations also rescued the temperature-sensitive phenotype of the psm3 ATPase mutant (*psm3-S1098A*) (Fig. 3c).

**Fig. 4.**
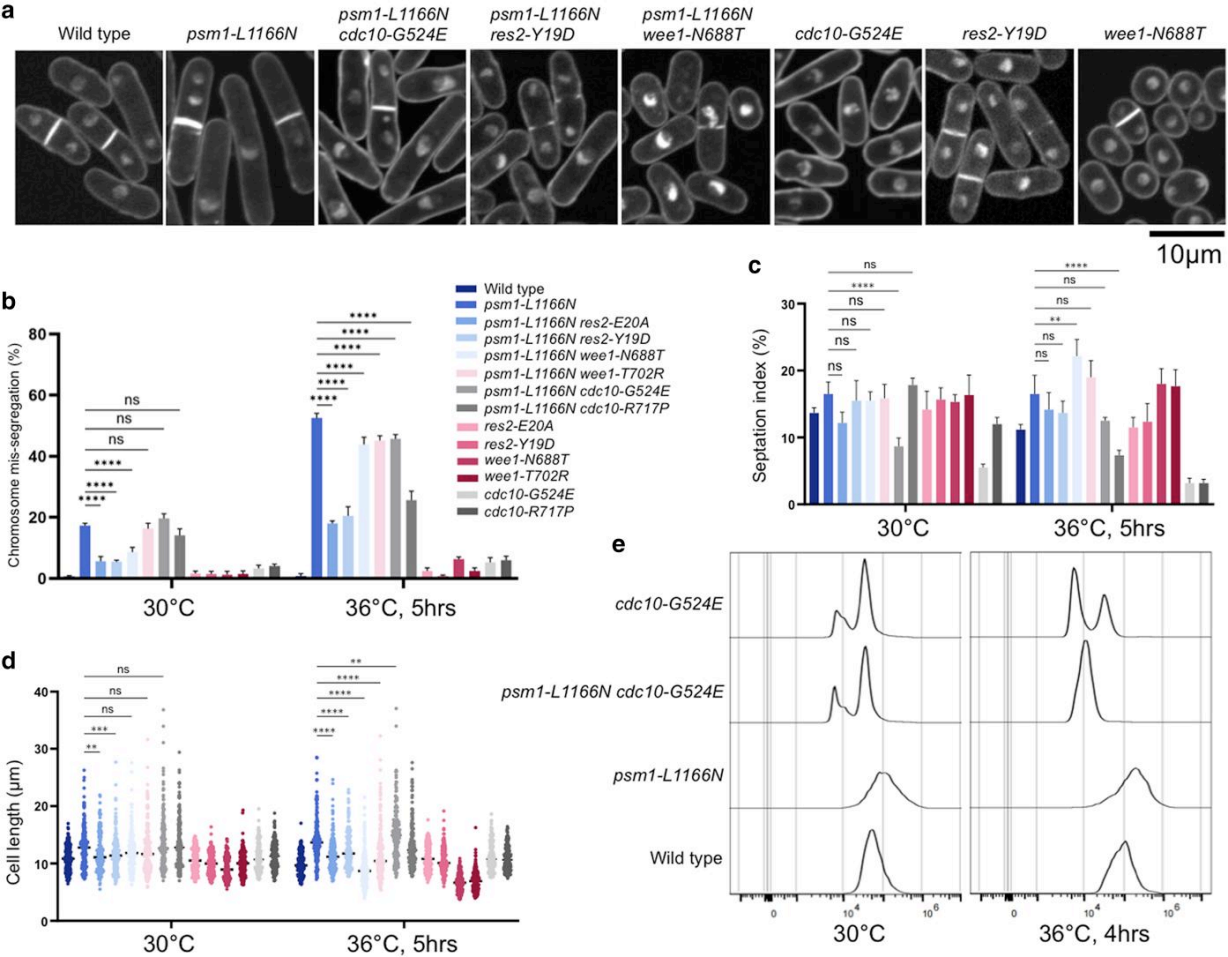
Phenotypic suppression of a cohesin ATPase mutant by mutations in Cdc10, Res2, and Wee1. a) Microscopic observation after DAPI staining of the mutants cultured at 36°C for 5 h. b) Frequency of chromosome mis-segregation events calculated by counting 200 mitotic cells. c) Septation index calculated by counting number of cells having septation in 200 cells. d) Cell length was measured using the ImageJ software. e) Flow cytometry analysis.

Microscopic analysis demonstrated that the *cdc10*, *res2*, and *wee1* mutations alleviated the chromosome mis-segregation and cell elongation phenotypes frequently observed in the cohesin ATPase mutants (*psm1-L1166N* and *psm3-S1098A*) (Fig. 4, a–d; Supplementary Fig. 2). Then, we performed flow cytometry analysis of the cell cycle progression for *cdc10* mutants. DNA content distribution indicated that peak of median fluorescence intensity of *cdc10* mutants (*cdc10-G524E* and *psm1-L1166N cdc10-G524E*) shifted to the left. It was reported that mutations in *wee1* endow cells with a significantly long G1 cell cycle interval (Singer and Johnston 1985). Therefore, mutations in these cell cycle regulators might cause cells having a longer G1 cell cycle interval (Fig. 4e; Supplementary Fig. 2d).

Cohesin binding to chromosomal DNA is dynamic in G1 phase and requires continued activity of the cohesin loader Mis4 (Bernard et al. 2008). Suppressor mutations of the 2 cohesin ATPase mutants were mapped either in Mis4's DBDs or in the cell cycle regulators, which indicated that extended G1 phase might enhance Mis4's binding to chromatin and further stabilize cohesin's association with chromatin.

### Mutations in Psm3 head domain rescue cohesin ATPase mutants

Suppressor mutations of the 2 cohesin ATPase mutants were also mapped in the 2 cohesin ATPase sites. These mutations were significantly enriched in the Psm3 ATPase sites (Psm3N and Psm3C) compared to those in the Psm1 ATPase sites (Psm1N and Psm1C) (Supplementary Fig. 3a). Localizations of suppressor mutations in Psm3N and Psm3C sequences were listed in Supplementary Fig. 3b and then compared with suppressor mutations of a cohesin loader ts mutant, *mis4-G1326E*. Some mutations were identified as suppressors of more than 1 ts mutant, such as the Psm3-I1153L mutation, which was identified as a suppressor of all the 3 ts mutants (*psm1-L1166N*, *psm3-S1098A*, and *mis4-G1326E*). The suppressor mutations of the 3 ts mutants (*psm1-L1166N*, *psm3-S1098A*, and *mis4-G1326E*) in Psm3 head domain and their overlap were presented in Supplementary Fig. 3c.

### *rad21-I67F* suppressors mapped in cohesin ATPase domains

**Fig. 5.**
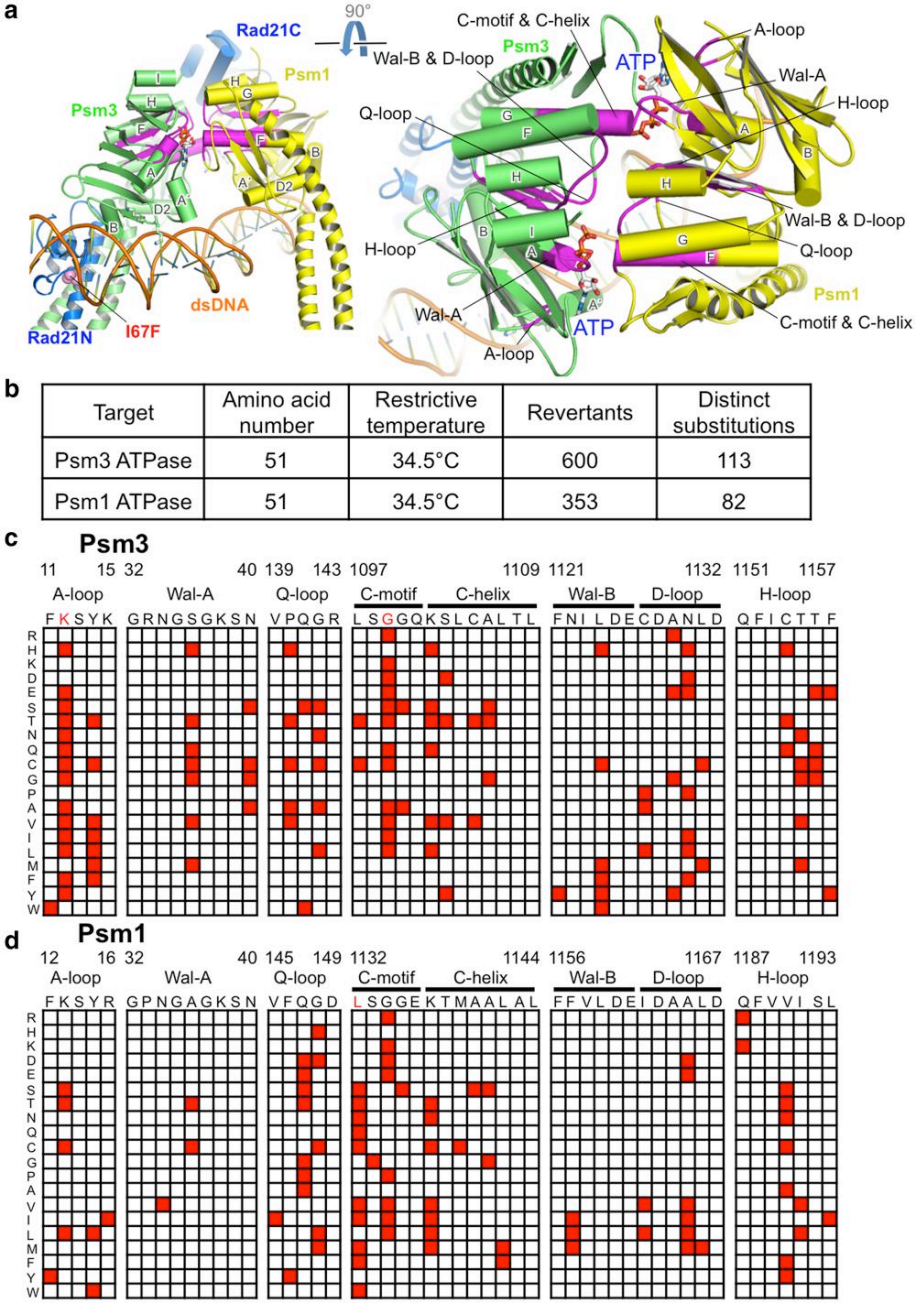
Mutations in cohesin ATPase domains rescued *rad21*. a) Structural view of cohesin head and locations of the conserved motifs in the 2 cohesin ATPase domains. A cohesin structure (PDB code: 6YUF) was used for presentation. Localization of the Rad21-I67F mutation was indicated too. b) Summary of the genetic screen, targeted to the 2 cohesin ATPase domains, for suppressor mutations that alleviated the premature cohesin releasing phenotype in *rad21-I67F*. c and d) Data matrices presenting single-amino-acid substitutions identified in Psm3 ATPase domain (c) or Psm1 ATPase domain (d) that rescued the temperature sensitivity of *rad21-I67F* at restrictive temperature.

*rad21-I67F* is a cohesin ts mutant that exhibits premature chromosome segregation phenotype at restrictive temperature (Tatebayashi et al. 1998). The responsible mutation, I67F, is located in the N-terminal domain (Rad21-NTD) of Rad21/SCC1 and may impair Rad21-NTD's interaction with Psm3/SMC3 coiled coils emerging from the head (Gligoris et al. 2014; Huis in ‘t Veld et al. 2014; Xu et al. 2018; Higashi et al. 2020) (Fig. 5a). A spontaneous suppressor screen for *rad21-I67F* was performed in a previous study and identified 5 suppressor mutations in the 2 cohesin ATPase domains (3 in Psm3 ATPase domain and 2 in Psm1 ATPase domain), which suggested that both Psm1 and Psm3 ATPase domains are required for cohesin releasing (Xu et al. 2018).

In this study, for each cohesin ATPase domain, we selected 51 amino acids located in conserved sequence motifs (Supplementary Fig. 1, c and d). Substitution of any of these amino acids would likely impair the corresponding ATPase domain's activity. We prepared a DNA library for each ATPase domain to mutate any 1 of the 51 amino acids to any other amino acids. The DNA libraries were independently transformed into the *rad21-I67F* ts mutant, followed by selection of survivors (revertants) at restrictive temperature (Tatebayashi et al. 1998; Tanaka et al. 2000). Targeted sequencing ultimately identified 113 distinct single-amino-acid substitutions in the Psm3 ATPase domain and 82 in the Psm1 ATPase domain that rescued the temperature sensitivity of *rad21-I67F* (Fig. 5b).

The single-amino-acid substitutions, which were identified as suppressors of *rad21-I67F*, were presented (Fig. 5, c and d). Figure 5c exhibited the suppressor mutations of *rad21-I67F* in Psm3 ATPase domain, while Fig. 5d exhibited the suppressor mutations of *rad21-I67F* in Psm1 ATPase domain. Suppressor mutations are distributed across all the sequence motifs, but show a bias toward specific residues (Fig. 5, c and d). Some amino acids emerged as hotspots, such as K12 in Psm3 A-loop, G1099 in Psm3 C-motif, and L1132 in Psm1 C-motif (the amino acids highlighted in red in Fig. 5, c and d). In contrast, most residues in Wal-A and Wal-B, in both Psm1 and Psm3 ATPase domains, are refractory of suppressors.

### Analogous mutations in the 2 cohesin ATPase domains

**Fig. 6.**
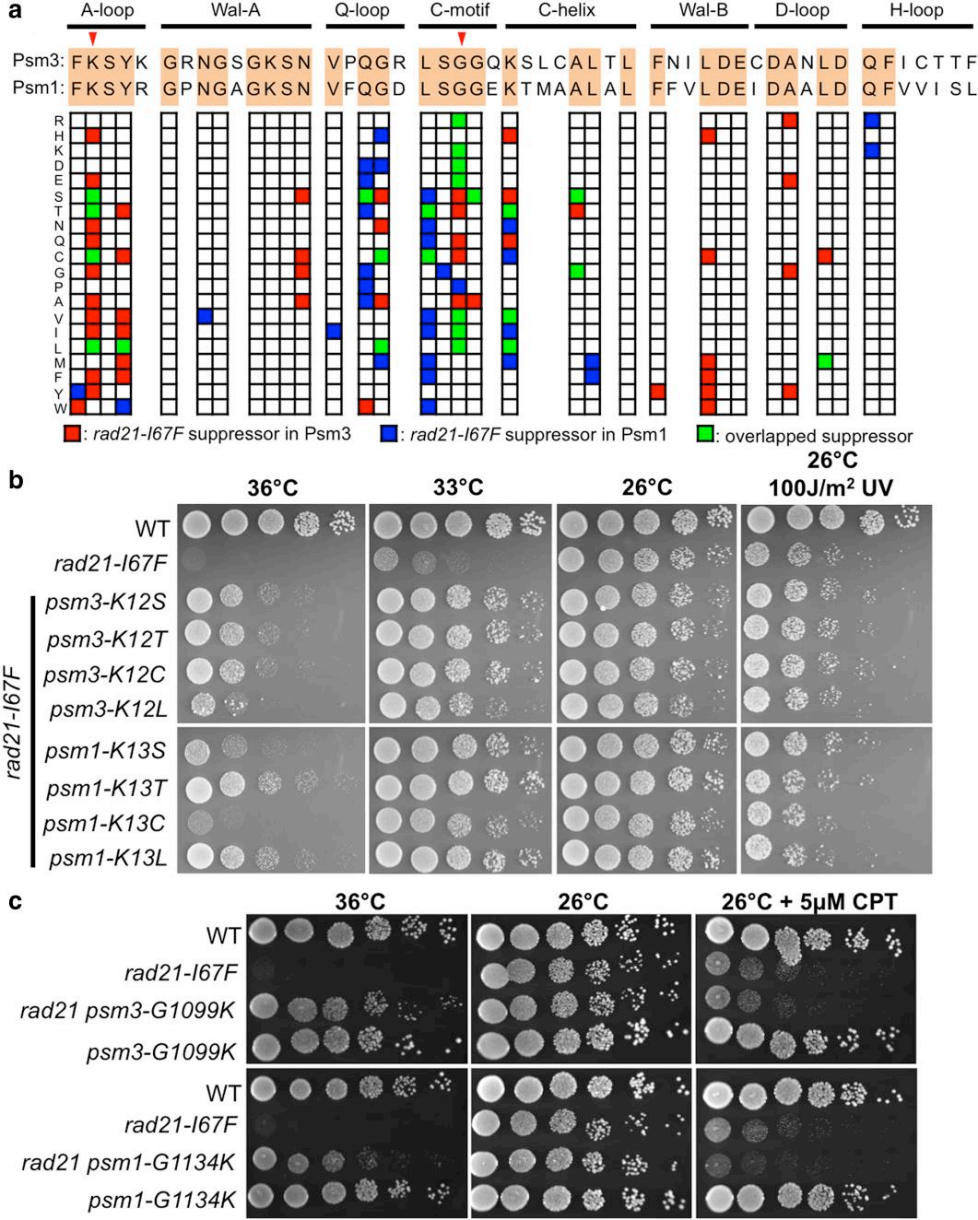
Analogous mutations identified in the 2 cohesin ATPase domains. a) Suppressor mutations of the *rad21-I67F* ts mutant, identified in the 2 cohesin ATPase domains, were presented in 1 data matrix. Locations of Psm3-K12 (and its corresponding amino acid Psm1-K13) in A-loop and Psm3-G1099 (and its corresponding amino acid Psm1-G1134) in C-motif were indicated by arrowheads. b) Spot test results of the analogous suppressor mutations at the conserved lysine in A-loop (Psm3-K12 corresponds to Psm1-K13). c) Spot test results of the analogous suppressor mutations at the conserved glycine in C-motif (Psm3-G1099 corresponds to Psm1-G1134).

The 2 ATPase domains of cohesin are highly conserved, and 32 of the 51 amino acids (62%), which were selected for mutagenesis, in each ATPase domain are identical. We compared suppressor mutations of *rad21-I67F* in Psm3 ATPase domain with those in Psm1 ATPase domain and found 21 identical single-amino-acid substitutions (analogous mutations). These analogous mutations were observed in A-loop, Q-loop, C-motif, C-helix, and D-loop, but most frequently in A-loop and C-motif (Fig. 6a), and especially at Psm3-K12 (and its corresponding amino acid Psm1-K13) of A-loop and Psm3-G1099 (and its corresponding amino acid Psm1-G1134) of C-motif (amino acids indicated by red arrowheads in Fig. 6a), as 4 and 7 identical single-amino-acid substitutions were observed, respectively. The data demonstrated that both Psm1 and Psm3 ATPase domains have similar roles in cohesin releasing. Spot test experiments indicated that the identical single-amino-acid substitutions observed in both Psm1 and Psm3 ATPase domains rescued the temperature sensitivity of *rad21-I67F* at similar levels (Fig. 6, b and c).

Cohesin's ATPase activities negatively regulate the interaction between Rad21's N-terminus and Psm3's coiled coils (Higashi et al. 2020; Muir et al. 2020). Suppressors of *rad21-I67F* (A Rad21 N-terminal mutant) were mapped to the conserved motifs in both cohesin ATPase domains (Xu et al. 2018). The unbiased and comprehensive genetic analysis performed in this study identified multiple analogous mutations in Psm1 and Psm3 ATPase domains that could rescue the growth defects of *rad21-I67F* at restrictive temperature. These findings suggested that both ATPase domains might negatively regulate the interaction between the Rad21's N-terminus and the Psm3's coiled coils. However, the *rad21-I67F* mutant's sensitivity to DNA damaging agents (ultraviolet or camptothecin) was not rescued (Fig. 6, b and c). These results indicated that the cohesion defects in *rad21-I67F* were rescued by suppressors in the cohesin ATPase domains, but the DNA repair defects in *rad21-I67F* were not.

### Suppressors of a coiled-coil mutant were mapped in coiled coils

**Fig. 7.**
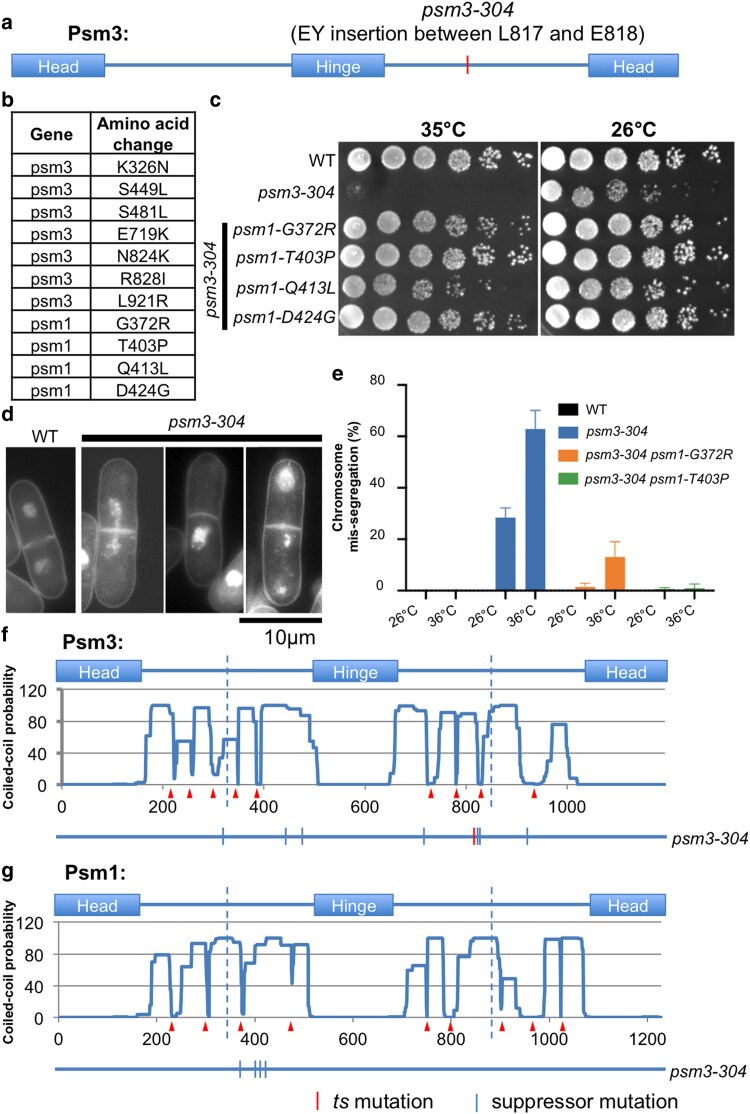
Suppressors of a Psm3 coiled-coil ts mutant identified in Psm1 and Psm3 coiled coils. a) The *psm3-304* ts mutant contains a 2 amino acids (“EY”) insertion in the Psm3 coiled coil between L817 and E818. b) Suppressor screen for the *psm3-304* mutant at restrictive temperature (37°C), followed by next-generation sequencing, identified mutations in Psm1 and Psm3. c) Spot test analysis. d) DAPI staining followed by microscopic observation for *psm3-304* mutant cells cultured at restrictive temperature 36°C for 5 h. e) Frequency of chromosome mis-segregation events calculated by counting number of cells exhibiting chromosome mis-segregation in 200 mitotic cells. f and g) Localization of the *psm3-304* suppressor mutations in the Psm3 and Psm1 protein sequences. The arrowheads demonstrated the positions of discontinuities (or breaks) that were predicted in the Psm1 and Psm3 coiled coils. The vertical dashed lines indicated the middle positions of the coiled coils.

Next-generation sequencing of a Psm3 ts mutant, *psm3-304*, identified an “EY” insertion in Psm3 coiled coil between amino acids L817 and E818 as the responsible mutation (Fig. 7a). Suppressor screen at restrictive temperature (37°C), followed by next-generation sequencing of revertants' genomic DNAs, identified multiple mutations in Psm1 and Psm3 (Fig. 7b). Re-integration of the suppressor mutations identified in Psm1 (G372R, T403P, Q413L, or D424G) into the *psm3-304* mutant confirmed the genetic suppression at restrictive temperature (Fig. 7c). The *psm3-304* mutant exhibited chromosome mis-segregation phenotype at restrictive temperature (Fig. 7d), and the suppressor mutations in Psm1 alleviated the chromosome mis-segregation phenotype greatly (Fig. 7e). Mutational localization analysis found that suppressor mutations of *psm3-304*, which were mapped in Psm1 and Psm3, were all located in the coiled coils of Psm1 and Psm3 (Fig. 7, f and g).

In addition, we performed a suppressor screen for a condensin coiled-coil temperature-sensitive mutant *cut14-208* (responsible mutation: S861P) at the restrictive temperature (34.5°C) (Sutani and Yanagida 1997), followed by next-generation sequencing of revertants' genomic DNA (Supplementary Fig. 4a). Two suppressor mutations in Cut14 were identified (Supplementary Fig. 4b). Spot test experiment confirmed the genetic suppression (Supplementary Fig. 4c). Mutational localization analysis revealed that the 2 suppressor mutations in Cut14 are located in the upstream coiled coil, whereas the original ts mutation, Cut14-S861P, is located in the downstream coiled coil of Cut14 (Supplementary Fig. 4d).

Overall, suppressor analysis for a cohesin (and condensin) mutant with the corresponding mutation in coiled coil identified multiple mutations also located in coiled coils. Since coiled coils are not continuous (containing breaks) and suppressor mutations are frequently located in or around these break sites, proper folding of the coiled coils might be critical for cohesin and condensin to fulfill their functions.

### Suppressors of a hinge interface mutant were mapped in the other hinge interface

**Fig. 8.**
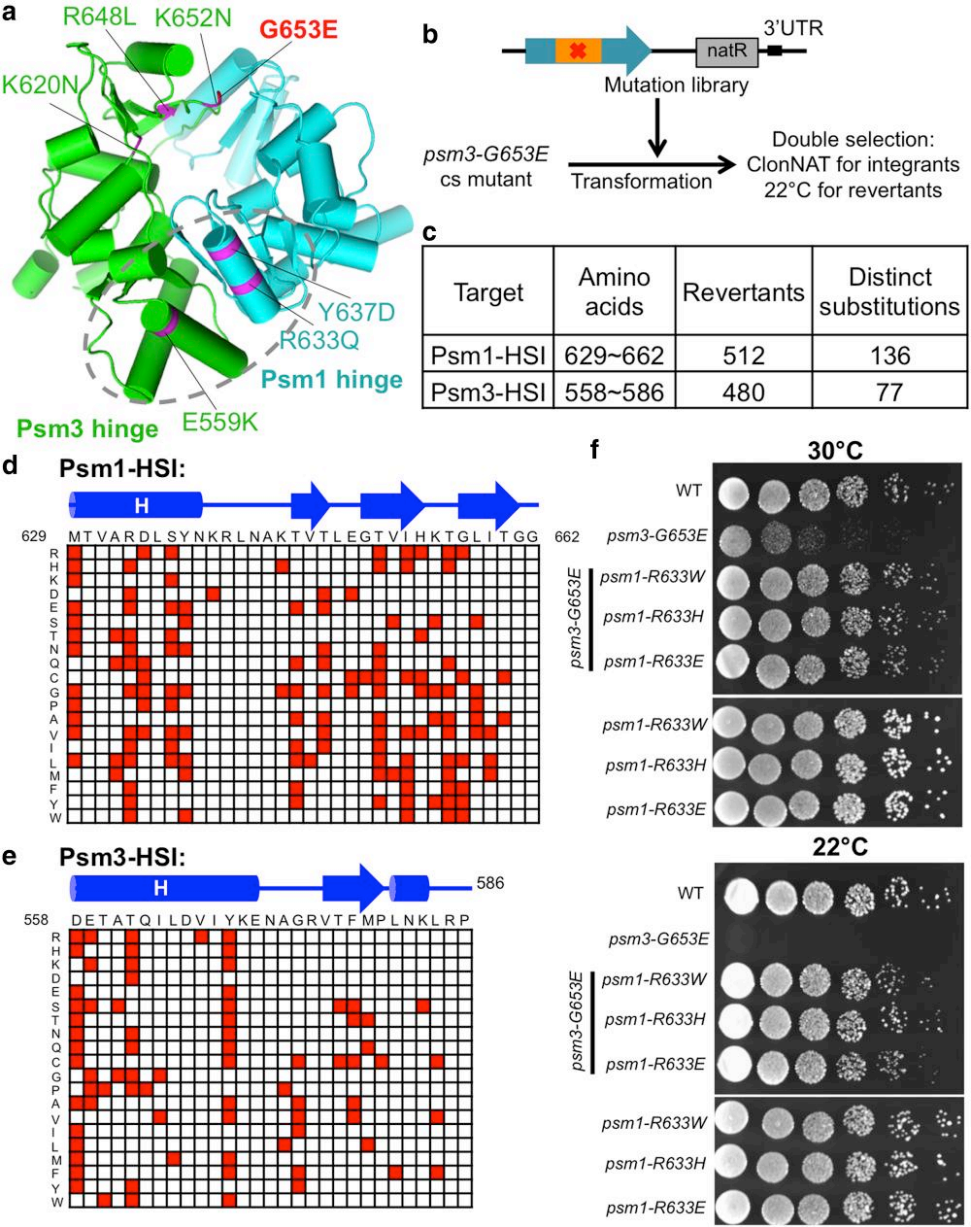
Suppressors of a Psm3 hinge interface mutant identified in Psm1 and Psm3 hinges. a) Suppressor mutations of a Psm3 hinge interface mutant, *psm3-G653E*, that were identified by next-generation sequencing. Revertants were isolated at restrictive temperature 22°C. b) Strategy of targeted mutagenesis, transformation, and revertant screening. c) Summary of the suppressor screen. d and e) Data matrices presenting the single-amino-acid substitutions identified in the Psm1 hinge domain d) or Psm3 hinge domain e) that rescued the cold sensitivity of *psm3-G653E*. f) Spot test analysis.

Cohesin Psm1 and Psm3 hinge domains interact to form a donut-like structure, which contains 2 interaction interfaces. The *psm3-G653E* mutant, isolated in a previous study, is sensitive to cold (20°C), and its responsible mutation, G653E, locates at 1 cohesin hinge interface (Xu et al. 2018) (Fig. 8a). Suppressor screen at restrictive temperature (22°C), followed by next-generation sequencing, identified multiple suppressor mutations. These mutations were enriched at the both cohesin hinge interaction interfaces (Fig. 8a). In this study, we performed serial targeted mutagenesis, aiming to mutate any one of the amino acids in the opposing hinge interface of Psm3-G653E to any of the other 19 amino acids (or stop codons) and then selected revertants which could alleviate the cold lethality of the *psm3-G653E* mutant at restrictive temperature 22°C (Fig. 8, b and c). Targeted sequencing of the mutated DNA regions identified more than 200 single-amino-acid substitutions (Fig. 8, d and e). Re-integration of the suppressor mutations in Psm1 hinge (Psm1-R633W, Psm1-R633H, or Psm1-R633E) into the *psm3-G653E* mutant confirmed the genetic suppression (Fig. 8f).

In addition, a suppressor screen for a condensin hinge interface ts mutant, *cut14-T558L* (responsible mutation: T558L) (Petrova et al. 2013), was performed at restrictive temperature 37°C. Then, next-generation sequencing identified multiple single-amino-acid substitutions covering the hinge domain (Supplementary Fig. 5a). Further spot test experiment verified the genetic suppression (Supplementary Fig. 5b).

Both cohesin and condensin hinges contain 2 distinct interaction interfaces. Suppressor screens for a hinge mutant, with corresponding mutation located in 1 hinge interface, identified multiple mutations in the other hinge interface. The phenomenon was observed for both cohesin and condensin hinge mutants. Therefore, the results indicated that the 2 hinge interfaces (in both cohesin and condensin) are functionally interconnected and work collaboratively in hinge–hinge interactions for their stable association with chromatin.

## Supplementary Material

jkaf111_Supplementary_Data

## Data Availability

The sequencing data reported in this paper have been deposited in the National Center for Biotechnology Information BioProject database (accession nos. PRJNA450289, PRJNA525996, and PRJNA846538). Supplemental material available at G3 online.
