# Supplementary material for: Site-to-site mutational dissection of fission yeast cohesin reveals its dynamics
Source: G3 (Bethesda). 2025 May 19;15(7):jkaf111. doi: 10.1093/g3journal/jkaf111 (PMC12239626; doi:10.1093/g3journal/jkaf111)
Supplement: jkaf111_Supplementary_Data [file jkaf111_supplementary_data.pdf]

Supplementary Information for

Site-to-site mutational dissection of fission yeast cohesin reveals its dynamics

Qi Wei[1*], Li Wang[2*], Yichen Zhang[1], Saidaiguli Abulimiti[1], Jie Wang[1], and Xingya Xu[1]†

[1]Institute of Future Agriculture, Northwest A&F University, Yangling, Shaanxi 712100, People's Republic of China.

[2]College of Enology, Northwest A&F University, Yangling, Shaanxi 712100, People's Republic of China.

[*]These authors contributed equally to this work

†Correspondence to:

Xingya Xu (xuxingya@nwafu.edu.cn)

This PDF file includes:

Figures S1 to S5

Table S1

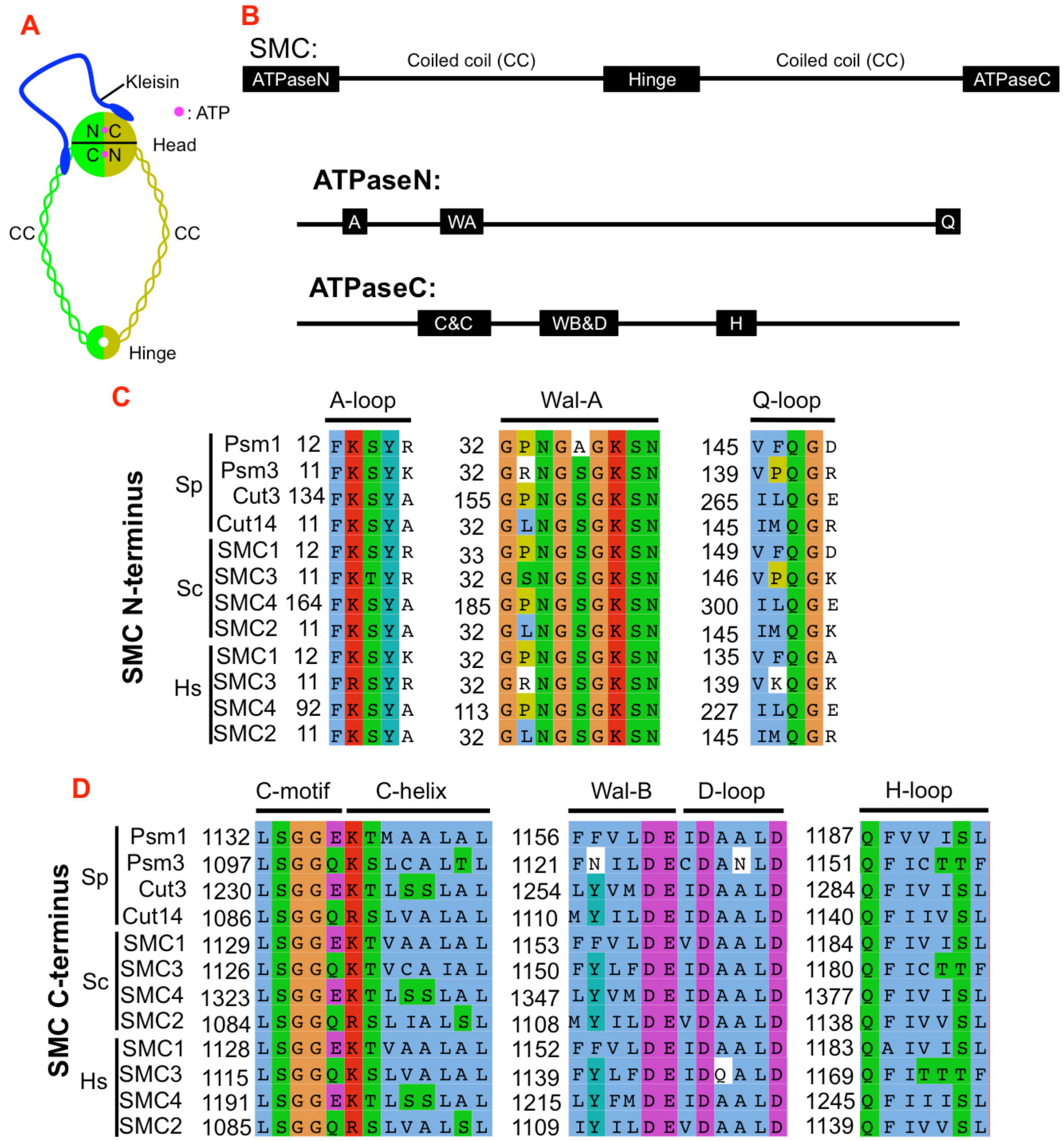

Figure S1. Cohesin organization and ATPase domains. (A) A schematic structure of cohesin. (B) The conserved motifs in the cohesin ATPase domains. SMC: Psm1 or Psm3; A: A-loop; WA: Walker A motif; Q: Q-loop; C&C: C-motif and C-helix; WB&D: Walker B motif and D-loop; H: H-loop. (C) Amino acid sequence alignment at sequence motifs of ATPase domains in N-terminal part of head domain. (D) Amino acid sequence alignment at sequence motifs of ATPase domains in C-terminal part of head domain.

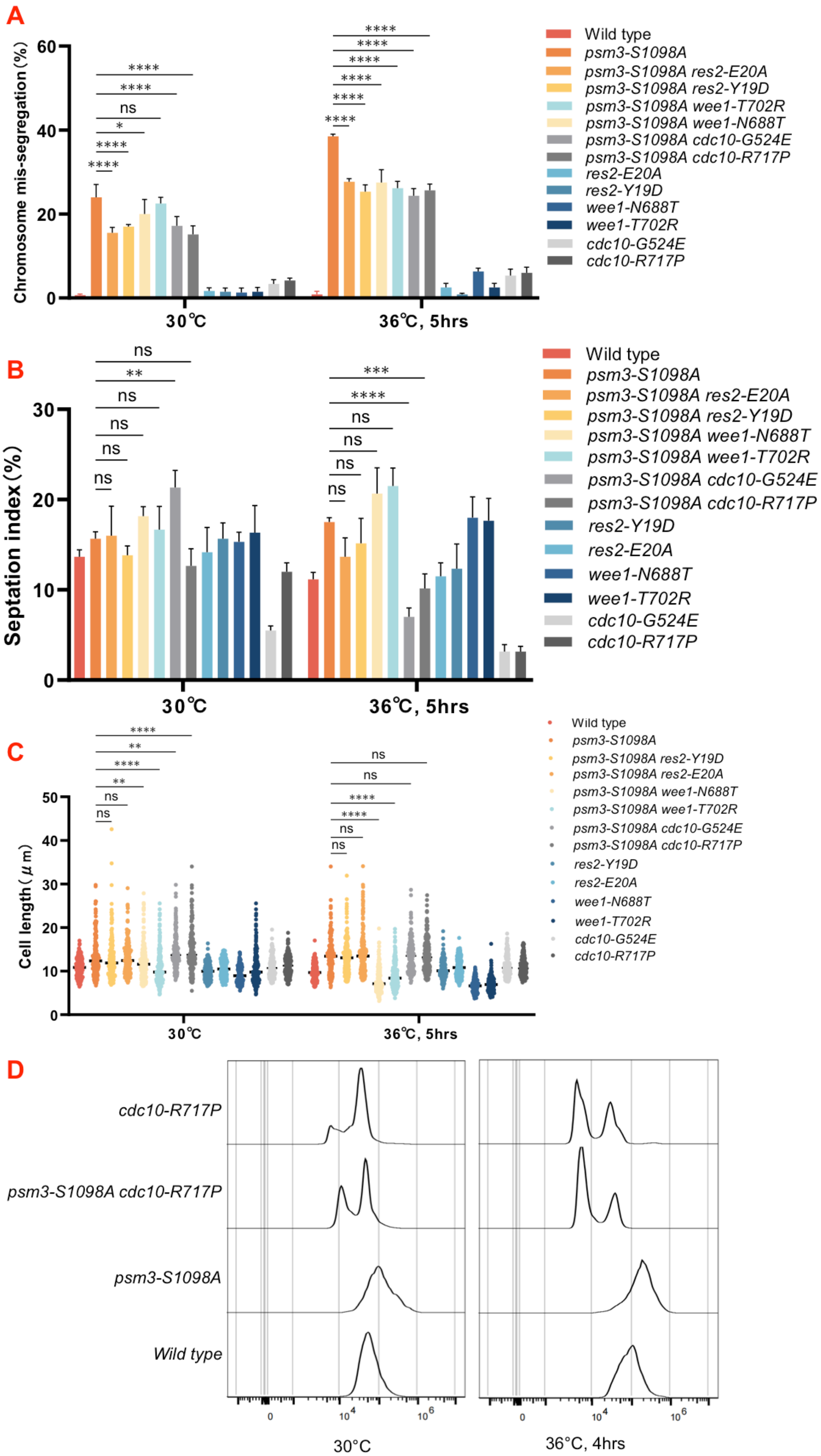
A
Chromosome mis-segregation (%)
0
20
40
60
****
****
*
ns
****
****
****
****
****
****
****
****
30℃
36℃, 5hrs
Wild type
psm3-S1098A
psm3-S1098A res2-E20A
psm3-S1098A res2-Y19D
psm3-S1098A wee1-T702R
psm3-S1098A wee1-N688T
psm3-S1098A cdc10-G524E
psm3-S1098A cdc10-R717P
res2-E20A
res2-Y19D
wee1-N688T
wee1-T702R
cdc10-G524E
cdc10-R717P
B
Septation index (%)
0
10
20
30
ns
**
ns
ns
ns
ns
***
****
ns
ns
ns
ns
30℃
36℃, 5hrs
Wild type
psm3-S1098A
psm3-S1098A res2-E20A
psm3-S1098A res2-Y19D
psm3-S1098A wee1-N688T
psm3-S1098A wee1-T702R
psm3-S1098A cdc10-G524E
psm3-S1098A cdc10-R717P
res2-Y19D
res2-E20A
wee1-N688T
wee1-T702R
cdc10-G524E
cdc10-R717P
C
Cell length (μm)
0
10
20
30
40
50
****
**
****
**
ns
ns
ns
ns
****
****
ns
ns
30℃
36℃, 5hrs
Wild type
psm3-S1098A
psm3-S1098A res2-Y19D
psm3-S1098A res2-E20A
psm3-S1098A wee1-N688T
psm3-S1098A wee1-T702R
psm3-S1098A cdc10-G524E
psm3-S1098A cdc10-R717P
res2-Y19D
res2-E20A
wee1-N688T
wee1-T702R
cdc10-G524E
cdc10-R717P
D
cdc10-R717P
psm3-S1098A cdc10-R717P
psm3-S1098A
Wild type
0
10^4
10^6
30°C
36°C, 4hrs

Figure S2 Phenotypic suppression of a Psm3 ATPase mutant by mutations in Cdc10, Res2 and Wee1. (A) Frequency of chromosome mis-segregation events calculated by counting 200 mitotic cells. (B) Septation index calculated by counting number of cells having septation in 200 cells. (C) Cell length was measured using the ImageJ software. (D) Flow cytometry analysis.

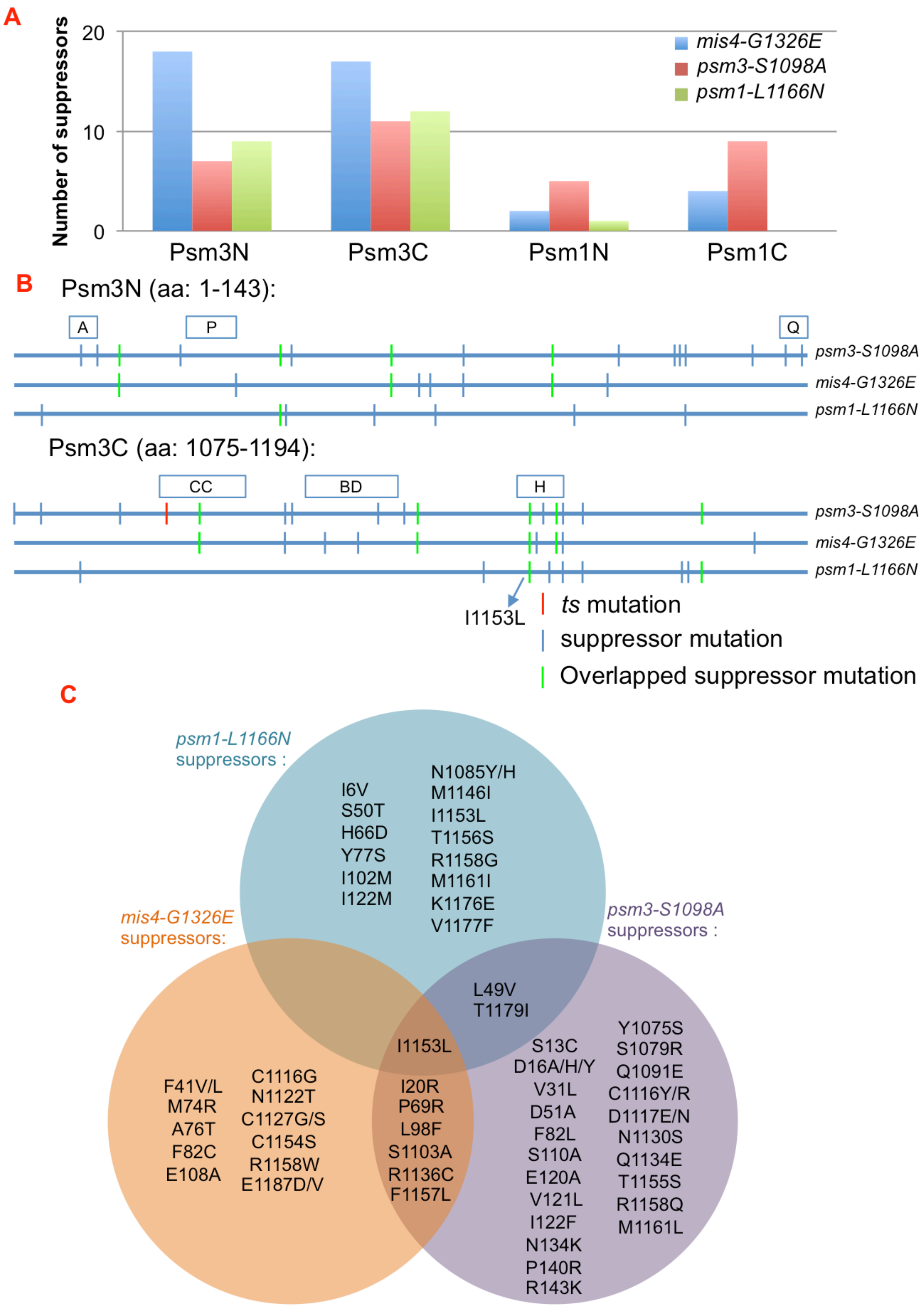

Figure S3 Extragenic suppressors of cohesin ATPase mutants in the Psm3 head domain. (A) Number of suppressors mutations of cohesin ATPase mutants,

identified in the Psm3 head domain (Psm3N and Psm3C) and the Psm1 head domain (Psm1N and Psm1C). (B) Localization of suppressor mutations of cohesin ATPase mutants identified in the Psm3 head domain. Bars indicated the localization of mutations. (C) Venn diagram of the suppressor mutations of the three ts mutants (*psm1-L1166N*, *psm3-S1098A* and *mis4-G1326E*).

A

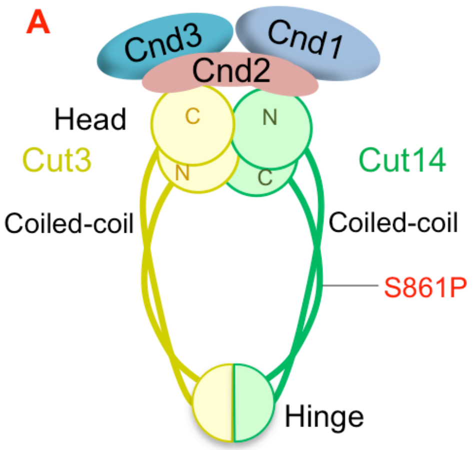

B

Gene	Amino acid change	Mutation
cut14	S315R	Chr II: 2880424:G->T
cut14	S371L	Chr II: 2880257:G->A

C

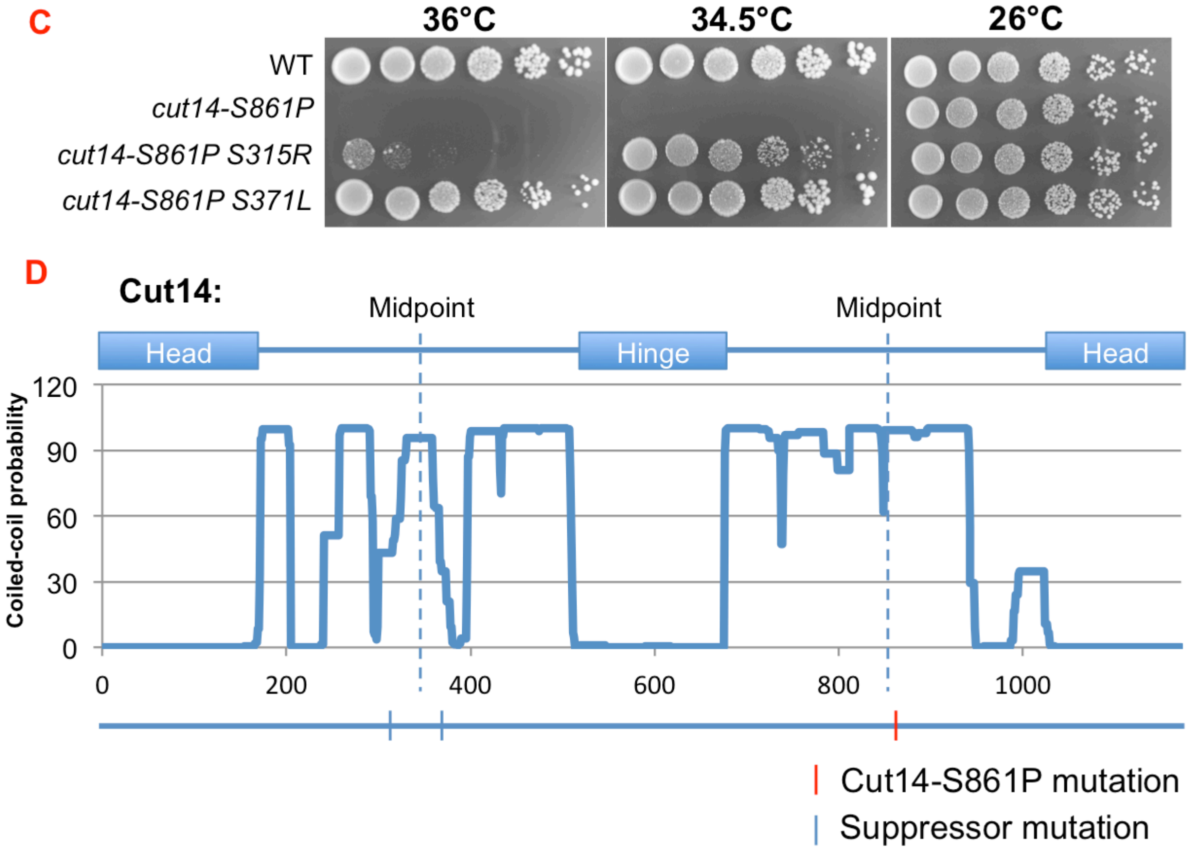

Figure S4 Suppressors of a condensin coiled coil ts mutant. (A) Representative localization of the responsible mutation, S861P, of a condensin coiled coil ts mutant, *cut14-208*, in condensin. (B) Suppressors of the *cut14-208* mutant identified by next-generation sequencing. (C) Spot test analysis. (D) Localization of the suppressor mutations of the *cut14-S861P* mutant in the Cut14 protein sequence.

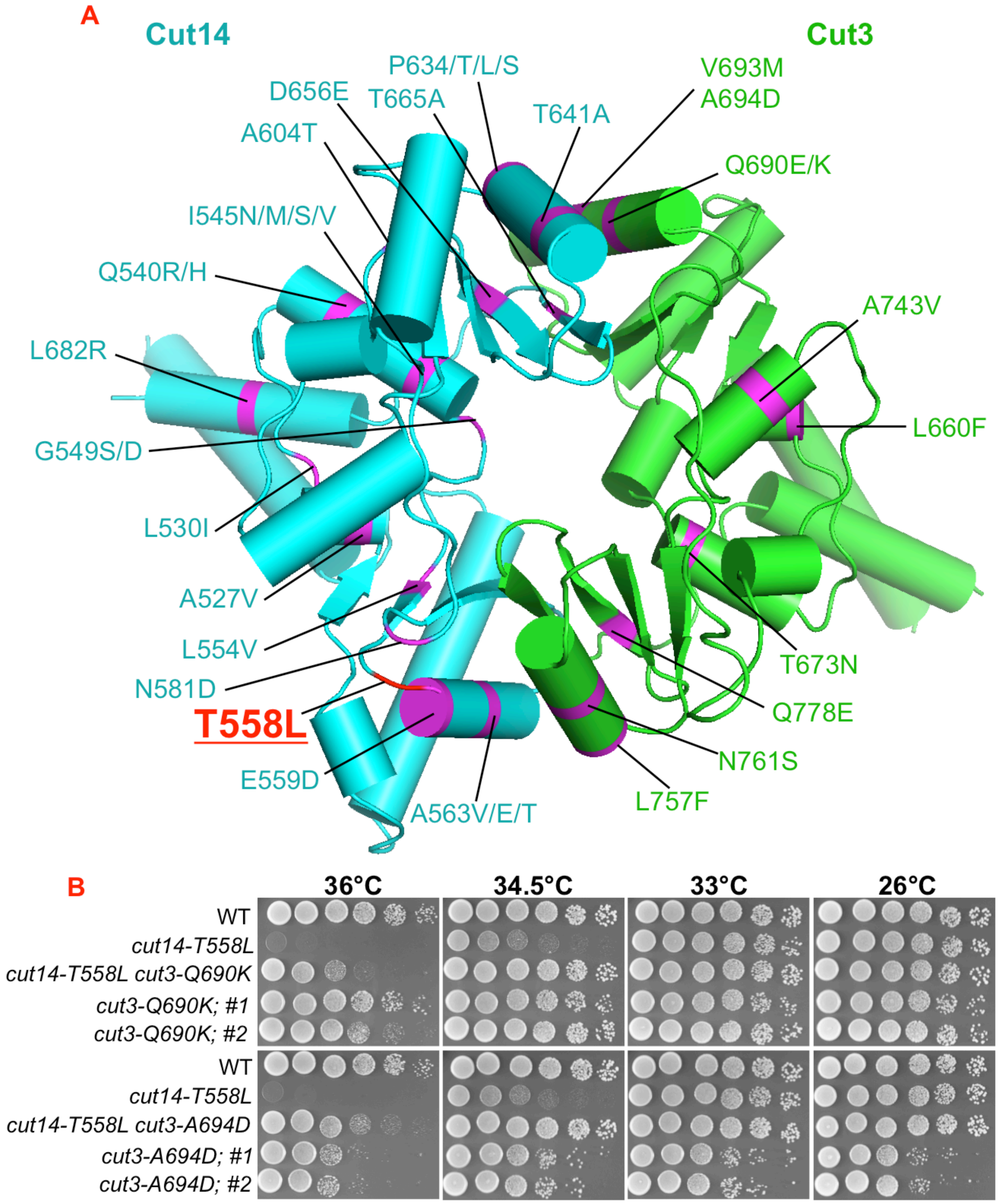

Figure S5 Suppressors of a condensin hinge interface ts mutant. (A) Suppressor mutations of a condensin Cut14 hinge interface mutant *cut14-T558L*, identified by next-generation sequencing. (B) Spot test analysis.

A

Suppressor gene	Amino acid change	Mutation	Original ts mutant
mis4	L346V	395772:G->C	*psm3-S1098A*
mis4	P810R	394292:G->C	*psm3-S1098A*
mis4	E818Q	394269:C->G	*psm3-S1098A*
mis4	I833K	394223:A->T	*psm3-S1098A*
mis4	D836G	394214:T->C	*psm3-S1098A*
mis4	Q878K	394089:G->T	*psm3-S1098A*
mis4	S903R	394012:A->C	*psm3-S1098A*
mis4	F1069L	393514:A->C	*psm3-S1098A*
mis4	R1116I	393374:C->A	*psm3-S1098A*
mis4	G1440W	392324:C->A	*psm3-S1098A*
mis4	I803M	394312:G->C	*psm1-L1166N*
mis4	T837A	394212:T->C	*psm1-L1166N*
mis4	D1439Y	392327:C->A	*psm1-L1166N*

B

Suppressor gene	Amino acid change	Mutation	Original ts mutant
res2	M1T	686594:T->C	*psm1-L1166N*
res2	Y13F	686630:A->T	*psm1-L1166N*
res2	Y19D	686647:T->G	*psm1-L1166N*
res2	E20A	686651:A->C	*psm1-L1166N*
res2	M29L	686677:A->T	*psm1-L1166N*
res2	A46G	686729:C->G	*psm1-L1166N*
res2	V69L	686797:G->C	*psm1-L1166N*
res2	V81F	686997:G->T	*psm1-L1166N*
res2	L89V	687021:C->G	*psm1-L1166N*
res2	L89Q	687022:T->A	*psm1-L1166N*
res2	E109Stop	687081:G->T	*psm1-L1166N*
res2	L201Stop	687358:T->G	*psm1-L1166N*
res2	E223Stop	687423:G->T	*psm1-L1166N*
res2	E224Stop	687426:G->T	*psm1-L1166N*
res2	H249R	687502:A->G	*psm1-L1166N*
res2	H253L	687514:A->T	*psm1-L1166N*
res2	V362G	687841:T->G	*psm1-L1166N*
res2	L490P	688225:T->C	*psm1-L1166N*
res2	-	688273:AT->A	*psm1-L1166N*
res2	Y589Stop	688523:T->G	*psm1-L1166N*
res2	K612Stop	688590:A->T	*psm1-L1166N*
res2	Y614Stop	688598:T->A	*psm1-L1166N*
cdc10	M1L	2763590:T->G	*psm1-L1166N*
cdc10	R717P	2761441:C->G	*psm1-L1166N*

C

Suppressor gene	Amino acid change	Mutation	Original ts mutant
res2	G15V	686636:G->T	*psm1-L1132T*
res2	V18F	686644:G->T	*psm1-L1132T*
res2	V56F	686758:G->T	*psm1-L1132T*
res2	-	686807:G->GATAT	*psm1-L1132T*
res2	A90G	687025:C->G	*psm1-L1132T*
res2	-	687046:G->GAATAATGAGCCC	*psm1-L1132T*
res2	M99R	687052:T->G	*psm1-L1132T*
res2	I102R	687061:T->G	*psm1-L1132T*
res2	Q119Stop	687111:C->T	*psm1-L1132T*
res2	-	687435:A->ATTCC	*psm1-L1132T*
res2	-	687458:GC->G	*psm1-L1132T*
res2	-	687487:T->TTGA	*psm1-L1132T*
res2	-	687499:GC->G	*psm1-L1132T*
res2	S288R	687618:A->C	*psm1-L1132T*
res2	L304R	687667:T->G	*psm1-L1132T*
res2	L307P	687676:T->C	*psm1-L1132T*
res2	L307R	687676:T->G	*psm1-L1132T*
res2	N363K	687845:T->G	*psm1-L1132T*
res2	A381P	687897:G->C	*psm1-L1132T*
res2	Y391Stop	687929:T->G	*psm1-L1132T*
res2	R400Stop	687954:C->T	*psm1-L1132T*
res2	S447Stop	688096:C->A	*psm1-L1132T*
res2	R473Stop	688173:C->T	*psm1-L1132T*
res2	-	688273:AT->A	*psm1-L1132T*
res2	Y525Stop	688331:C->G	*psm1-L1132T*
res2	-	688565:CA->C	*psm1-L1132T*
res2	T607P	688575:A->C	*psm1-L1132T*
cdc10	P308S	2762669:G->A	*psm1-L1132T*
cdc10	A492E	2762116:G->T	*psm1-L1132T*
cdc10	N514H	2762051:T->G	*psm1-L1132T*
cdc10	G524E	2762020:C->T	*psm1-L1132T*

D

Suppressor gene	Amino acid change	Mutation	Original ts mutant
wee1	P406H	723450:C->A	*psm1-L1132T*
wee1	H626D	724109:C->G	*psm1-L1166N*
wee1	L674V	724253:T->G	*psm1-L1166N*
wee1	N688T	724296:A->C	*psm1-L1166N*
wee1	T702R	724338:C->G	*psm1-L1166N*

Table S1 Suppressor mutations of the cohesin ATPase mutants identified by next-generation sequencing
